# Forecast analysis of the incidence of tuberculosis in the province of Quebec

**DOI:** 10.1186/1471-2458-13-400

**Published:** 2013-04-27

**Authors:** Alexander Klotz, Abdoulaye Harouna, Andrew F Smith

**Affiliations:** 1 Medmetrics Inc, Suite # 215, 925 De Maisonneuve Avenue West, Montreal, QC H3A 0A5, Canada; 2 Adjunct Professor (Health Economics), McGill University, Montreal, QC, Canada

**Keywords:** Tuberculosis, Epidemiology, Forecasting, Quebec

## Abstract

**Background:**

While the overall population prevalence of tuberculosis in Quebec has been declining for many years, tuberculosis is still disproportionately more prevalent among the immigrant and Inuit communities. As such, the aim of this study was to forecast the incidence of tuberculosis in the Province of Quebec over time in order to examine the possible impact of future preventative and treatment programs geared to reducing such disparities.

**Methods:**

A compartmental differential equation based on a **S**usceptible **E**xposed **L**atent **I**nfectious **R**ecovered (SELIR) model was simulated using the Euler method using Visual Basic for Applications in Excel. Demographic parameters were obtained from census data for the Province of Quebec and the model was fitted to past epidemiological data to extrapolate future values over the period 2015 to 2030.

**Results:**

The trend of declining tuberculosis rates will continue in the general population, falling by 42% by 2030. The incidence among immigrants will decrease but never vanish, and may increase in the future. Among the Inuit, the incidence is expected to increase, reaching a maximum and then stabilizing, although if re-infection is taken into account it may continue to increase. Tuberculosis among non-indigenous Canadian born persons will continue to decline, with the disease almost eradicated in that group in the mid 21st century.

**Conclusions:**

While the incidence of tuberculosis in the Province of Quebec is expected to decrease overall, certain populations will remain at risk.

## Background

Tuberculosis (TB), which is caused by the bacteria *Mycobacterium tuberculosis* is an infectious disease which predominantly infects the lungs. Canada has one of the lowest incidences of TB in the world, and rates have been falling in Canada since the 1970s [1]. Tuberculosis rates in the Province of Quebec have been lower than the Canadian average since 1986, but increased rates are seen amongst certain populations, notably the Inuit and immigrants [2]. The epidemiological trends of tuberculosis in Quebec were discussed in a systematic review by Klotz *et al*. [3], who found that the incidence is decreasing among the general and immigrant populations but may show an increase among the Inuit. This paper concentrates on attempts to model and forecast the incidence of tuberculosis among these at risk groups to inform future treatment and prevention programs.

The rate of tuberculosis is approximately ten times higher in immigrants compared to Canadian-born persons, in part due to immigration from countries with high levels of TB such as Haiti and India [2]. In the Province of Quebec, the greatest incidences are among the Inuit, where they can approach 50 times that of the general population [2]. While incidences are high, the low numbers of Quebec’s Inuit population mean that there are often less than 10 cases per year. In many Nunavik (Northern Quebec) communities, for example, the lack of adequate medical facilities necessitates flying infectious individuals to the nearest hospital, greatly increasing the cost of diagnosis and treatment. The rate among non-Inuit aboriginal populations is higher than non-Indigenous Canadian-born citizens, but overall numbers in this group are low and are not discussed in this analysis.

While historical data can be used to gauge the present situation and observe trends, it does not provide immediate knowledge of the future of the disease. To predict the epidemiology of a disease in the future, mathematical models must be employed. Epidemiological modelling of disease, including tuberculosis, is an established practice. In this regard, the first mathematical model of tuberculosis was developed by Waaler in 1962 [4], which used an ordinary differential equation to forecast tuberculosis infection. Although other mathematical methods exist, differential equation models are a ubiquitous tool. A review paper by Colijn *et al*. [5] summarises the history and functionality of many of the models which have been previously employed. The differential equation models generally divide the population into compartments, for example, **s**usceptible, **i**nfectious, and **r**ecovered (SIR). There are demographic parameters such as birth and death rates as well as the natural history of infection parameters such as the rate of infection that determine the relative behaviour of these groups. A paper by Garcia *et al*. [6] describes a more complex model, with compartments for vaccination, latency, and chronicity. The model used in this paper is similar to one used by Castillo-Chavez [7] and attempts to take into account the overall behaviour of the disease while ignoring some of the more complicated aspects such as vaccination.

## Methods

**Figure 1:**
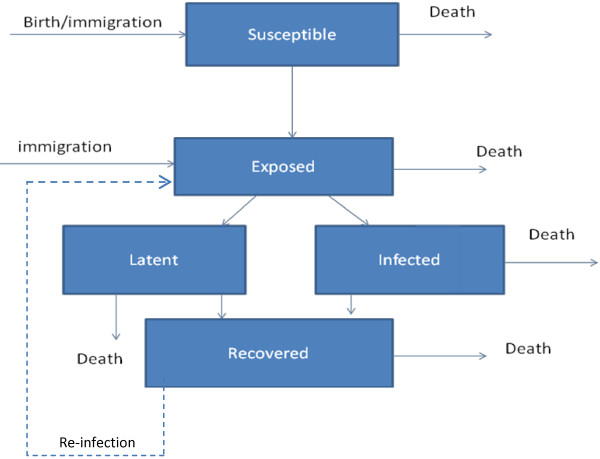
Flow chart of the model.

An ordinary differential equation was used to model the spread of tuberculosis throughout the population of the Province of Quebec. A compartmental model was developed with five categories: **S**usceptible, **E**xposed, **L**atent, **I**nfectious, **R**ecovered, known as a SELIR model (Figure 1). People were born into the S category with a birth rate b, and people from all categories died with death rate μ. People progress from the susceptible to exposed category through contact with infectious people, accounted for by an interaction term and a transmission factor, β, equivalent to the number people a single infectious person exposes yearly. Exposed individuals are removed from that group at a rate k, and become infectious or latent with a probability of α and 1- α, respectively. Yearly, infectious and latent individuals have a probability γ_2_ and γ_1_ of recovering, respectively. These recovery parameters are based on current epidemiological data and thus take into account current treatment methods. The coupled differential equations that make up the model are thus:

$$\frac{\mathrm{dS}}{\mathrm{dT}}=b\left(1-q\right)N-\mathrm{\mu S}-\mathrm{\beta S}\frac{I}{N}$$

$$\frac{\mathrm{dE}}{\mathrm{dT}}=\mathrm{bqN}-\mathrm{\mu E}+\mathrm{\beta S}\frac{I}{N}-\mathrm{kE}$$

$$\frac{\mathrm{dL}}{\mathrm{dt}}=\left(1-\alpha \right)\mathrm{kE}-\mathrm{\mu L}-\gamma_{1}L$$

$$\frac{\mathrm{dI}}{\mathrm{dt}}\mathrm{akE}-\mathrm{\mu I}-\gamma_{2}I$$

$$\frac{\mathrm{dR}}{\mathrm{dt}}=\gamma_{1}L+\gamma_{2}I-\mathrm{\mu R}$$

Values for parameters were selected based on various estimates and published values and are described in Table 1.

**Table 1:** Definitions and values of parameters

**Symbol**	**Meaning**	**Typical value**	**Source**
b	Birth/immigration rate	0.03/person/year	[11]
μ	Death rate	0.007/person/year	[11]
β	Transmission Parameter	5/person/year	Fitting
q	Proportion of exposed immigrants	0.01	[2]
α	Probability of infectiousness	0.1	[5]
K	Rate of progression	0.05/person/year	[6], [12]
γ_1_	Rate of recovery (latent)	0.22/person/year	[5]
γ_2_	Rate of recovery (infectious)	0.87/person/year	[5], [2]

Greek letter parameters were subject to a sensitivity analysis varying them by up to 25%.

The model was modified to account for immigration. Instead of a birth rate adding to the population of susceptibles, a constant immigration rate added the majority of the new population to the susceptible group, but a small fraction q to those exposed. This is designed to mirror the fact that each year there is a sub-population of new immigrants who have been exposed to TB. Because most cases last for less than a year, the number of cases per capita can serve as a proxy for the order of magnitude of the incidence of the disease. Thus, the fraction of exposed immigrants was estimated as ten times the incidence per capita of immigrants in the Province of Quebec using 2009 data. This is based on the fact that, according to Colijn [5] 10% of those exposed to the disease will eventually become infectious. If we treat each new yearly population of immigrants as the source for N potential cases, then approximately 10 N would have been exposed. The infection parameter, β, was estimated by using a least-squares algorithm to fit the model to published epidemiological data. The populations that are known at the beginning of the simulation are only the number of infections in a given year, and the total population. The smoothness of the dynamics depends on an appropriate ratio of exposed to infectious persons, which is not known *a priori*. A heuristic estimate can assume, based on Colijn [5], that for every infectious case there are nine latent cases, and that based on the progression rate of one twentieth per year, the number of exposed people should be of order 180 times that of infectious, with some variation. To better estimate this, we fit this ratio to the epidemiological data and find that a value of 167 was consistent with the disease in the Province of Quebec.

If the initial numbers of the various populations are not consistent with what would emerge naturally from this model, there is a rapid increase or decrease of their ratios in the first few years of simulation. To guarantee smoothness, the fits were pre-seeded, running for six years before being fit to the data, such that the initial ratios were consistent with the dynamics of the model. This ensured that the ratio of exposed to infectious persons was stable over long time scales, and that any discontinuities occur before the fitting and forecasting began. In order to propagate the uncertainties in the fits to historical data, two Gaussian random number distributions were generated with means and standard deviations taken from the regression of the infection parameter and the exposed:infectious ratios. The distributions were used to forecast the disease into the future, using 200 iterations of the model to generate a range for the prediction, establishing upper and lower bounds for the spread of disease. We performed a sensitivity analysis using Latin hypercube sampling and found that the model is robust under variations of the parameters, with the model being most sensitive to the infectiousness ratio α.

The parameter that is used as an output from the simulations is the number of new cases per year, as this is the common form of the available epidemiological data and serves as the best comparison between model and reality. It can also be used to examine the financial burden of the disease in an economic extension of the model.

The model was simulated using Euler’s method with a time-step of one month (the results were compared to those of the Runge–Kutta method and found to be identical). This was implemented using MATLAB for quick development as well as Visual Basic for Applications (VBA), interfaced through Microsoft Excel. The latter implementation, while not the fastest, was chosen to allow the program to be used by public health professionals who are not familiar with computer programming. This is part of a longer-term project [3] to aide public health professionals who are concerned with disease progression, who will be able to use the program to input various epidemiological information and quickly ascertain the long-term effects. The program used Excel as a graphical interface, with rate constants and initial values entered as inputs on a spreadsheet and read by the VBA module. The output was displayed on the same interface.

## Results

Data from Canada [1] and the Province of Quebec [2] and government reports on tuberculosis were used. A cursory examination of the data shows that the rates are generally decreasing. With data spanning an interval from 1985 to 2009, it is possible to fit the model in a simple case assuming a homogeneous population. The total output over the 24 year period can be used to heuristically validate the model: if the disease has been accurately described by SELIR dynamics, the same dynamics will continue to model the spread of the disease.

There were 586 cases in the Province of Quebec in 1985 and 195 in 2009. Overall, the fit of the model to the data was found to be high (R^2^=0.92). Fitting to the data, the background exposure level was found to be 168 times the infectious level, and a transmission parameter of 4.0±0.4 was found. This is consistent with a similar model by Castillo-Chavez [7] used to model the disease in the United States: within the context of their model, they find a value of 2.2. It should be noted that the definition of the β parameter varies from model to model so direct comparisons are unwise. The model can then forecast beyond 2009 to an arbitrary date in the future. We predict, for example, that the number of new cases in the Province of Quebec in 2025 will be roughly 150. However, this is not meant to be an actual prediction, but rather an example data set for examining the model. We can also compare the model to a much simpler one, for example, exponential decay: a fit of the SELIR model to the first half of the data is more accurate at predicting the second half of the data than is exponential decay.

**Figure 2:**
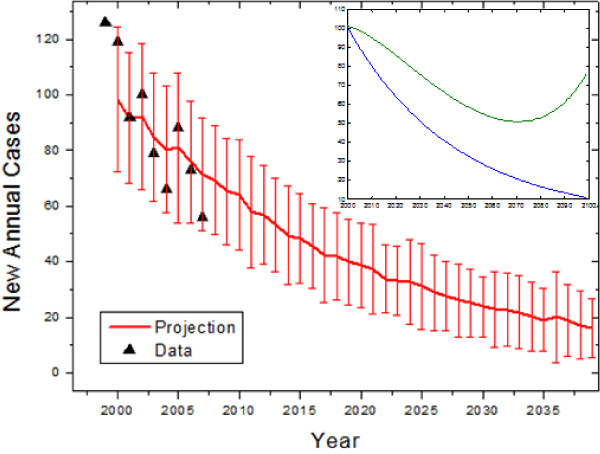
**Retroactive and future predictions of the model for the Canadian-born non-indigenous Quebec population**. Error bars represent standard deviations of forecast distributions. Inset: Comparison of mean long term trend treating the Canadian-born and immigrant populations separately (blue), and with full interaction (green).

Although not the primary target of tuberculosis interventions, the model was used to forecast the data in non-indigenous people of Canadian birth. Tuberculosis among this group has been decreasing over the past several years, and is in fact consistent with exponential decay (R^2^=0.85). Fitting the model to the data, we found that the decay of new infections was consistent with a lower bound of zero transmissions (R^2^=0.56). Forecasting into the future using these results, we expect that there will be fewer than 30 new cases yearly by 2030 (Figure 2).

**Figure 3:**
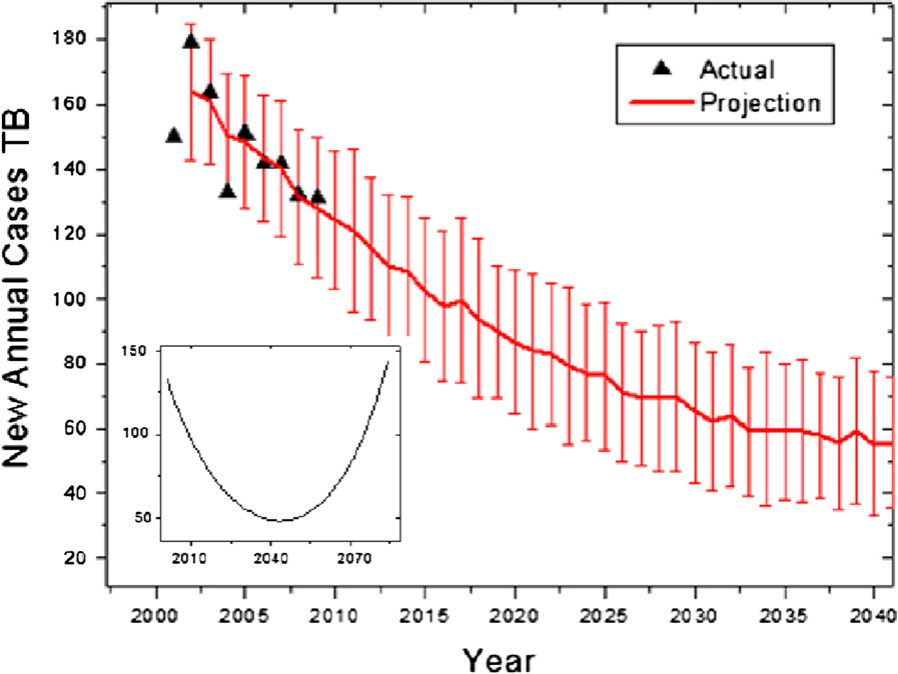
**Retroactive and future predictions of the model for the immigrant population.** Error bars represent standard deviations of forecast distributions. Inset: Long-term prediction, showing the eventual rise in the late 21st century.

The fitting for the immigrant model begins with the 2001 census for the Province of Quebec, which reported an immigrant population of 706,975 and 37,604 new immigrants that year, while 150 new cases among immigrants were reported. An unknown, but important parameter is the percentage of exposed immigrants. We estimate this to be 130 exposed per 100,000 new immigrants yearly, based on 2009 epidemiology data. Using the 2001 through 2009 data as a basis, we found a best-fit (R^2^=0.68) transmission parameter of 2.72 ± 1.98 and an initial exposed to infectious ratio of 167, both consistent with our estimates for the homogeneous population. The model predicts a slow decline in the disease, with the median prediction dropping below 100 cases within several years (Figure 3). However, due to the fact that diseased individuals continue to seed the exposed population, the disease will not be eradicated among immigrants. We found that over long enough times frames, the number of new annual cases will begin to increase in the middle of the century (Figure 3, inset).

The Inuit population of the Province of Quebec is largely isolated [8], but this is not the case for the immigrant and Canadian-born populations. The immigrant and Canadian populations were modelled separately because transmission is known to largely occur within immigrant communities [9]. However, this assumption may be challenged given that many immigrants in Quebec live in Montreal, the largest city, and are integrated into the general public. If the two populations are treated together (allowing transmission from one group to the other), the immigrant population is not noticeably affected by interacting with the Canadian-born, but the yearly decrease in cases is expected to slow among the Canadian born (Figure 2, inset), reaching a local minimum around 2070 before rising again, while in the worst-case scenario the number of predicted cases with interaction would double that without interaction by 2060. However, within the time span of interest, such an interaction in the worst case would increase the total number of cases by about 10%.

**Figure 4:**
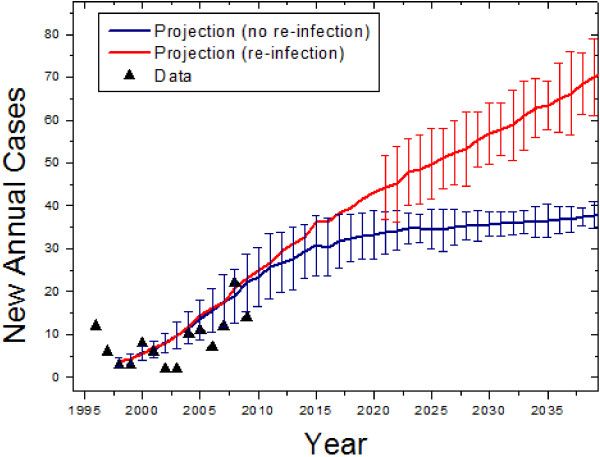
**Retroactive and future predictions of the model for the Inuit Quebec population.** Error bars represent standard deviations of forecast distributions. The red curve represents the prediction when re-infection (a transfer of recovered persons to the exposed category) is taken into account, and the error bars are suppressed at early times as a visual aide.

The Inuit population can be difficult to model epidemiologically because of its low population: minor variations in cases can have large effects on the overall trend. The Inuit population of the northern region of the Province of Quebec was 7,765 in 1996 according to the Circumpolar Statistics Database [10], and there were 12 new TB cases according the Quebec government [2]. Epidemiological data from the years 1998 to 2009 were used in order to obtain the best fit parameters (R^2^=0.64). The transmission parameter was found to be much higher than among immigrants: 61.90 ±16.3. The exposed to infectious ratio was found to be 216. The validation and forecast for the Inuit population can be seen in Figure 4, with (red) and without (blue) re-infection taken into account.

Defining upper and lower bounds on the predictions, we found that the disease will increase to a plateau of about 30 cases in the 2020’s.

Epidemiological reports indicate that re-infections were responsible for 6% of total cases in Quebec in 2000 [3]. While this fraction is small overall, the higher prevalence among the Inuit may suggest that it plays a larger role. We examined the role of re-infection in the Inuit with minimal assumptions: that the re-transmission rate is the same as the initial transmission rate, and that the disease proceeds identically after re-infection. Colijn *et al*. [5] demonstrated that re-infection was important if the rates of re-infection were similar to those of initial infection. The modification to the model to include this is represented by the dashed line in Figure 1. Simulating the Inuit population taking this into account, the initial pattern of the disease is the same except the number of new cases continues to grow, rather than plateauing (See Figure 4). In the immigrant and non-indigenous populations, re-infection has negligible effect; taking it into account does not change the error bounds of the prediction and changes the mean by less than a percent. Overall, we found that the number of new tuberculosis cases in non-Indigenous people of Canadian birth will continue to decrease, that it will decrease in the short term but not permanently in immigrants, and that it will increase among the Inuit. A summary of our forecasts can be found in Table 2.

**Table 2:** Summary of model predictions

**Year**	**Canadian born**	**Immigrants**	**Inuit**	**Total**	**Drop from 2009**
2015	48 (16)	102 (22)	30 (7)	181 (28)	16%
2020	38 (15)	86 (22)	33 (6)	158 (27)	26%
2025	24 (15)	76 (22)	35 (5)	141 (28)	34%
2030	19 (11)	64 (22)	36 (3)	124 (24)	42%

Parentheses represent bounds on predictions.

## Discussion

The numerical results of our simulations are presented in Figures 2, 3, 4. Overall, we found that the general declining trend in the incidence of tuberculosis in the Province of Quebec will continue, especially as seen heuristically when the population is modelled as homogeneous. This will be particularly rapid among non-indigenous Canadians, while immigrant incidence rates will decrease at a slower rate. As the rate of TB in immigrants and the general population declines, the contribution to the total number of cases by the Inuit will increase.

The predictions for immigrant cases show that the disease will not vanish. The upper bound on the transmission rate predicts that the number of immigrant cases may start to increase in the 2040s. This is consistent with the predictions of Zhou *et al*. [11] from 2008: who predict that an increase in immigrant cases will cause an increase in the non-immigrant cases by 2012, although a re-analysis with recent data may delay that prediction. The slower decline and subsequent increase in immigrant cases is in part due to the proportion of exposed immigrants that arrives each year and in part due to the greater transmission factor compared to the Canadian-born population. The greater transmission factor may be due, in part, to the tendency of immigrant communities to live in closer proximity to one another as in Montreal, as described by Haase *et al*. [9]. The low socioeconomic status of immigrants from high-incidence countries may also contribute to this. The results of our forecast show that if measures are not taken to screen immigrants for tuberculosis and to reduce the transmission of the disease between immigrants, the trend may reverse mid-century and TB may become more prevalent. Given that immigrant populations are a major contributor of tuberculosis cases, the future of the disease in Canada should be examined in context of future increased immigration.

The high transmission parameter and predicted increase for the Inuit may appear alarming, but the growth is not expected to continue indefinitely and in the long term the per capita incidence disease will be present but stable. This is similar to the findings of Blower *et al*. [12], who predict a plateau in the relative proportion of infections over long durations. Nevertheless, the number of new annual cases among the Inuit is expected to increase. Re-infection may play a significant role as suggested by our simulations, as the higher proportion of both recovered and infectious persons makes contact more likely. If these predictions are accurate, the costs of diagnosing and treating the disease, which are higher in the northern regions of the Province of Quebec due to low density populations and necessity of air travel, may increase.

More data may be needed to understand the long-term trends in this population and a more complicated model than a homogeneous differential equation may be necessary to take into account such fluctuations. Nguyen *et al*. [13], for example, noted that the transmission was based on interactions between different villages in Nunavik. With small populations, stochasticity in transmission may play a role. Epidemiological models such as those taking into account stochastic processes [14] in disease progression may be more accurate in describing the Inuit population over long time spans.

It may be of interest for future researchers to study in greater depth the dynamics of this particular model, beyond short- and medium-term predictions for Quebec. Future work can examine it in the context of broader analyses of tuberculosis dynamics in low incidence-high immigration countries [15,16]. However, such analyses were deemed to be beyond the scope of this paper because they are not expected to lead to short-term changes in the forecasts. Long term trends can be examined (as appear on insets in Figures 2 and 3), but over many decades they will lose validity because the demographic models have constant birth, death, and immigration rates leading to exponential population growth. Over longer time periods, changes to these rates and the onset of logistic growth are expected to occur and more assumptions are required to model the demographics.

It is of note that the latent category in this model does not explicitly have an effect on the dynamics: it merely serves as another compartment for exposed individuals. When re-infection is taken into account there is the possibility of people in the latent category becoming infectious after recovery. Although the model may be simplified by removing the transition to latency, it is necessary for future work in which the financial cost of latent tuberculosis, on the order of several hundred dollars [17] per patient, must be taken into account. If re-infection is included, as discussed previously, there is now an opportunity for latent individuals to become infectious.

Certain occurrences may reduce the validity of these results, for example, if at some time in the future a new treatment for TB is discovered and implemented, or a new deadlier or more drug-resistant strain breaks out. The predictions of this model would no longer adequately describe those situations, although modifications to the recovery parameters may account for such developments. To incorporate such an effect in the model, a parameter can be modified at a specific time (for example, the recovery parameter would be increased in the case of a better drug) which would manifest itself as a cusp on the forecast curve. However, predicting such changes are beyond the scope of this paper.

The simulations described in this paper are part of a larger study that serves to estimate the future financial burden of tuberculosis [3]. Among the non-indigenous Canadian-born population the burden will be minimal, as the disease slowly becomes less and less common. The results of these simulations, however, indicate that without intervention, tuberculosis among immigrant populations in the Province of Quebec will not vanish, and incidence of the disease will slowly increase among the Inuit. These may lead to greater expenditure required to control the disease, if public health policy is not modified to take into account tuberculosis within these two groups.

While it may appear that these results are specific to the Province of Quebec, they highlight the challenges of disease management in the 21st century. The Province of Quebec is an example of an affluent industrialized society with a large immigrant population and a disadvantaged indigenous population. In this respect, the epidemiology of tuberculosis between these three groups is similar in other Canadian provinces as well as other nations with similar population structures including the United States, Australia, New Zealand and South Africa as well as European nations with high rates of immigration. The forecasts presented herein may serve as a case study for a situation that may arise worldwide in the coming decades.

## Conclusions

A compartmental differential equation model was used to forecast the yearly incidence of tuberculosis for three at risk populations in the Province of Quebec. Tuberculosis has been declining in the Province of Quebec for many years, but is more prevalent among the immigrant and Inuit communities. Historical epidemiological data was used to verify the model, which was then used to forecast the rates for three population groups. It was found that the disease is vanishing among non-indigenous Canadian born persons, that the incidence is expected to decrease among immigrants but not vanish, and that among the Inuit a short term increase and subsequent leveling is predicted. Overall, the number of new annual cases will decrease.

## Competing interests

Alexander Klotz, Abdoulaye Harouna and Andrew F. Smith were employees of Medmetrics Inc at the time this research was conducted. The authors have no other competing interests.

## Authors’ contributions

AK: AK provided data analysis and literature searching skills. AK worked on the statistical analysis and interpretation of data flowing from the review of the literature and analyzed data on the trends of tuberculosis in the Province of Quebec. Lastly, AK participated in the drafting of the article reporting on the findings of this research. AH: AH contribution to this paper was in the form of providing support with the literature searches, extracting the data for analysis, assisting with the analysis of underlying trends in the data and in assisting with the drafting the manuscript reporting on the findings. AFS: AFS was responsible overall for obtaining funding for this research, the original conceptualization of the research aims, underlying methodological approach, devising the literature search strategy, analyzing the data, as well as drafting, editing and critically evaluating the intellectual content of the final manuscript. All authors read and approved the final manuscript.

## Pre-publication history

The pre-publication history for this paper can be accessed here:

http://www.biomedcentral.com/1471-2458/13/400/prepub
